# In the underbelly of grief

**DOI:** 10.1017/S1478951525101363

**Published:** 2025-12-29

**Authors:** Paul Rousseau

*Missing someone you love is hard, but never being able to see them again is harder.*

*Anonymous*

She died distant from home, in a medical mecca where everyone can be helped and everyone can be saved, except they can’t and they aren’t. Eventually, the flesh weakens, the breath wheezes, the heart rests.
She died quietly save a low guttural moan rolling up from her belly. I collapsed like a wilted sunflower bowing to the presence of death. There was sadness and weeping and wailing, but there was nothing to be done – she was dead, her body a cold husk of decomposing pulp.
And now, I wander untethered like a tree without roots. There is only longing and wanting and despair and grief. And grief, the painful promise of love, is nothing more than memories of what was, and what will never be again. It’s like being buried alive, unable to breathe, with no way out.

